# 3D-Printed Closed-Channel Spiral Inertial Microfluidic Device for Size-Based Particle Separation

**DOI:** 10.3390/mi17040435

**Published:** 2026-03-31

**Authors:** Eda Ozyilmaz, Gamze Gediz Ilis

**Affiliations:** 1 Department of Mechanical Engineering, Gebze Technical University, Kocaeli 41400, Türkiye; ggediz@gtu.edu.tr; 2 Institute for NanoSystems Innovation (NanoSI), Northeastern University, Boston, MA 02115, USA

**Keywords:** microfluidic, particle separation, inertial microfluidics, spiral microchannels, 3D printing, closed-channel microfluidics

## Abstract

Spiral inertial microfluidic devices provide a simple, high-throughput approach for size-based particle separation; however, translating PDMS-optimized designs into monolithic, fully enclosed 3D-printed channels is often limited by printability and post-print channel clearing. In our previous PDMS study, a 400×120µm spiral achieved high separation performance after computational optimization and experimental validation. To translate this high-performing PDMS concept into a faster and more cost-effective manufacturing approach, the same separation principle is transferred to a fully 3D-printed, closed-channel spiral device, and the geometry is re-optimized around manufacturability constraints. Printing trials showed that enclosed channels at 400×120µm and 600×180µm could not be cleared reliably due to trapped resin and frequent blockage, most often near the inner-outlet region. In contrast, 800×240µm and 1200×360µm channels were printed and flushed successfully, and 800×240µm was selected as the smallest reproducibly functional cross-section. Particle-tracking simulations were then used to re-optimize spiral development length, showing that a 4-turn device provides limited collection for 12µm targets (10%), intermediate lengths (5–7 turns) improve collection to 50%, and an 8-turn spiral achieves complete large-particle collection (100%) across tested target sizes (12–24µm) while reducing small-particle crossover. Experimental validation of the 8-turn 800×240µm device at $Q=6\;mL\;\min^{-1}$ using fluorescent polystyrene particles (18µm target; 6µm background) yielded an average collection efficiency of 84% and an inner-outlet purity of 92%. Overall, these results demonstrate that spiral inertial separation can be retained in a monolithic 3D-printed format when the design is re-optimized around the smallest reliably clearable enclosed cross-section and sufficient spiral development length.

## 1. Introduction

Microfluidic technologies enable precise manipulation of small-volume samples and have become a key platform for analytical and biomedical workflows due to their compact footprint, reduced reagent consumption, and integration potential for sample preparation and downstream analysis [1,2]. A central capability in many lab-on-a-chip systems is size-based particle and cell separation, which enables label-free enrichment and fractionation in continuous flow [3]. Although active methods such as dielectrophoresis, acoustics, and magnetophoresis can provide strong selectivity, they require external field sources and additional instrumentation that increase system complexity and can limit scalability [4,5]. In contrast, passive hydrodynamic approaches, particularly inertial microfluidics, have attracted wide interest for high-throughput, continuous, and label-free separation [6,7,8,9].

Inertial microfluidics exploits hydrodynamic lift forces and secondary flows that arise at moderate Reynolds numbers, driving particles toward size-dependent equilibrium positions [10,11]. In straight channels, migration is governed by the balance between shear-gradient-induced and wall-induced lift components, leading to stable focusing locations. In curved channels such as spirals, centrifugal effects generate transverse secondary flows (Dean vortices), and the resulting Dean drag interacts with inertial lift to enhance size-dependent lateral migration [12,13]. Spiral inertial microchannels are therefore widely used for size-based sorting because they combine compact footprint with operational simplicity and scalable throughput [14,15]. However, separation performance remains strongly coupled to geometry and operating conditions, including cross-sectional dimensions, curvature, flow rate, and spiral development length, motivating systematic design exploration rather than heuristic tuning [16,17].

In our previous study, this design coupling was addressed by computationally optimizing a PDMS-based spiral inertial microfluidic device using multiphysics simulations and an efficient experimental strategy. The optimized PDMS configuration used a 400×120µm rectangular cross-section and achieved high separation performance experimentally, highlighting the strong influence of geometry-driven parameters on focusing behavior and outlet partitioning [18]. Despite these advantages, translating PDMS-optimized designs into scalable manufacturing can be challenging due to multi-step processing, bonding variability, and limitations in forming robust, monolithic fluidic interfaces for repeatable deployment.

Additive manufacturing has emerged as a versatile fabrication route for microfluidic devices because it enables rapid prototyping, reduced assembly steps, and greater geometric flexibility compared with conventional manufacturing approaches. Among the commonly used 3D-printing technologies, fused deposition modeling (FDM/FFF) is attractive because of its low cost and ease of use, although its relatively low resolution and visible layer lines limit its suitability for fine microfluidic features. Vat-photopolymerization methods such as stereolithography (SLA) and digital light processing (DLP) are more relevant to microfluidics because they provide smoother surfaces and higher resolution through layer-by-layer curing of photopolymer resins. In particular, DLP offers faster printing than SLA by curing an entire layer at once while maintaining relatively high feature detail [19]. Other advanced additive-manufacturing methods, including selective laser sintering (SLS), Multi Jet Fusion (MJF), PolyJet, binder jetting, and direct metal laser sintering/selective laser melting (DMLS/SLM), have also been explored for specialized applications; however, their material limitations, post-processing demands, high cost, or lower suitability for enclosed transparent microchannels often reduce their practicality for routine microfluidic fabrication. Therefore, among currently accessible approaches, resin-based photopolymerization methods, especially SLA and DLP, have become the most promising platforms for fabricating enclosed microfluidic devices with improved dimensional fidelity and surface quality [20]. Additive manufacturing offers an attractive route for rapid iteration and monolithic integration of microfluidic devices with embedded connectors and complex geometries [21]. Vat photopolymerization methods (DLP/SLA) can produce transparent parts suitable for optical interrogation; however, fully enclosed microchannels introduce practical constraints beyond nominal printer resolution. In particular, reliable post-print clearing of long, narrow, enclosed channels is often limited by trapped uncured resin and local constrictions, which can lead to partial or complete blockage. As a result, geometries that perform well in soft lithography may not be directly transferable to monolithic, closed-channel printed devices without redesign around manufacturability.

In directly 3D-printed inertial or spatial separation devices, enclosed channel dimensions are often larger than those used here. For example, Sierra Agudelo et al. reported low-cost 3D-printed spiral inertial microfluidic devices with channel heights of 600 µm and later 800 µm to mitigate clogging, with widths of approximately 1.4 mm [22]. Other stereolithographic studies have demonstrated fully enclosed single-piece microchannels with high geometric complexity, but often at substantially larger channel heights or using specialized fabrication platforms. For example, Gong et al. reported a 41 mm long serpentine enclosed channel and high-aspect-ratio channels below 25 µm in width but 3 mm in height using a custom DLP-SLA system [23], while Kuo et al. demonstrated stacked and overlapping enclosed 3D microchannel architectures using a high-precision PEG-DA-258-based stereolithographic resin system, with representative channel heights on the order of 300–1000 µm depending on geometry [24]. By contrast, the 240 µm channel height and relatively small enclosed cross-section used in the present study represent a substantially smaller scale for a monolithic, fully enclosed printed spiral device. This is important because, in stereolithographic microfluidics, the challenge is not only to print small features but also to effectively remove uncured resin and preserve an open, functional channel after printing. In practice, manufacturability depends not only on channel height but on the overall enclosed cross-sectional size that governs resin drainage and post-print clearing [25]. Motivated by the need to translate a high-performing PDMS spiral separation concept into a faster and lower-cost fabrication route, this work demonstrates a fully enclosed 3D-printed spiral device fabricated by a low-cost DLP process and re-optimized around manufacturability constraints.

**Figure 1:**
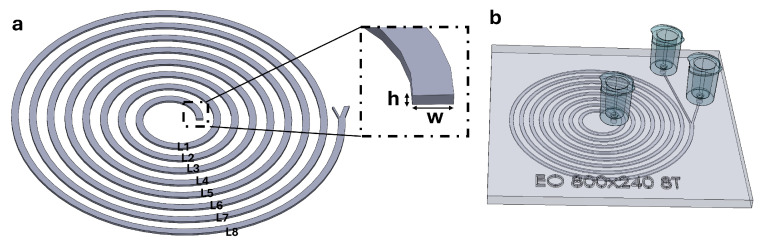
Spiral device concept and geometrical definitions. (**a**) 8-turn spiral model used for numerical simulations. (**b**) Monolithic 3D-printed closed-channel spiral device with two outlets (inner/outer).

In this work, a spiral inertial separation concept is translated from a PDMS prototype to a fully 3D-printed, closed-channel device by explicitly incorporating manufacturability into the design process. The PDMS-scale enclosed cross-section (400×120µm) and a moderately scaled variant (600×180µm) were first attempted, but these geometries could not be cleared reliably due to frequent blockage in enclosed features. The channel cross-section was therefore increased, and 800×240µm was identified as the smallest reproducibly functional enclosed geometry in the workflow, while 1200×360µm was also evaluated as a larger, robust-printing reference. Because scaling the cross-section changes the balance of inertial lift and Dean-flow-induced transport, spiral development length was re-optimized through turn-number variation to recover effective focusing and outlet segregation. Figure 1 summarizes the baseline spiral concept and the corresponding monolithic 3D-printed closed-channel implementation used in this study. Using fluorescent polystyrene particles as size-defined surrogates, the selected geometry and operating condition are experimentally validated, and separation performance is quantified in the printed platform. Collectively, this study provides an engineering pathway for translating optimized spiral inertial separators into manufacturable, monolithic 3D-printed closed-channel devices.

## 2. Materials and Methods

### 2.1. Spiral Channel Inertial Forces

Inertial microfluidic separation in spiral channels is governed by the interplay of hydrodynamic forces acting on suspended particles at moderate Reynolds numbers. Two primary force components determine the lateral equilibrium position of a particle within the channel cross-section: the inertial lift force, which arises from the parabolic velocity profile of the carrier fluid, and the Dean drag force, which originates from the curvature-induced secondary flow in spiral geometries [10,12,13]. The balance between these forces drives size-dependent lateral migration, enabling continuous and label-free particle separation. The governing expressions for each force component are summarized below.

Shear Gradient Lift: Directed from regions of high shear gradient (near the wall) toward regions of lower shear gradient (toward the channel centerline). The mechanism involves pressure differences generated by the curvature of the velocity profile pushing the particle toward the center.

Wall Induced Lift: Directed away from the wall, toward the centerline. The mechanism involves flow compression near the wall, creating a pressure imbalance that repels the particle from the wall.

Net Inertial Lift: Directed toward an equilibrium position located roughly 20–30% of the channel width from the wall.

(1) $$F_{L}∼C_{L}\;\rho \;U_{m}^{2}\;\frac{a^{4}}{D_{h}^{2}}$$

where $C_{L}$ is the lift coefficient (on the order of 0.5), ρ is the fluid density, $U_{m}$ is the mean flow velocity, *a* is the particle radius, and $D_{h}$ is the hydraulic diameter. Note that $F_{L}\propto a^{4}$, so lift rapidly decreases for small particles.

Dean Drag Force: The curvature of the spiral induces a pair of counter-rotating secondary vortices (Dean vortices). The drag exerted by this secondary flow on the particle is the Dean drag force.

(2) $$F_{D}\;\approx \;3\pi \;\mu \;a\;U_{\mathrm{Dean}}\;=\;3\pi \;\mu \;a\;U_{m}\sqrt{\frac{D_{h}}{2R}}$$

where μ is the fluid viscosity,

(3) $$U_{\mathrm{Dean}}=U_{m}\sqrt{\frac{D_{h}}{2R}}$$

is the Dean velocity, and *R* is the local radius of curvature of the spiral. Since $F_{D}\propto a$, Dean drag remains relatively significant for smaller particles compared to lift.

Focusing Criterion: It is well known that the particle-to-channel hydraulic diameter ratio must satisfy the following condition for effective inertial focusing [10]:

(4) $$\frac{a}{D_{h}}>0.07$$

Fabricating features that satisfy this ratio is only feasible using lithography or highly precise printing methods such as two-photon polymerization (2PP) [26]. Therefore, compensating for the $a/D_{h}$ requirement by other means—such as increasing the spiral development length—is the most economical and practical solution in the present work.

Reynolds Number: Inertial focusing relies on a balance between inertial lift forces and Dean secondary forces, both of which depend on the channel Reynolds number:

(5) Re=ρUavgDhμ

### 2.2. Device Design

A planar spiral inertial microfluidic separator with a rectangular cross-section (width *W*, height *H*) and two collection outlets (inner and outer) was used throughout this study. The spiral geometry used for (i) numerical simulations (Figure 1a) and (ii) monolithic 3D printing (Figure 1b) was designed in SolidWorks 2024 (Dassault Systèmes, Waltham, MA, USA) and exported as a 3D CAD model for numerical analysis and printing. For consistent post-processing, the spiral was indexed loop-by-loop (L1–L8) to visualize particle distributions at successive turns and to directly compare development-length effects between 4-turn and 8-turn designs.

A manufacturability-driven redesign was implemented by varying the channel cross-section and spiral development length (turn number) while screening size-based separation between a small/background particle population and a large/target population (Table 1). Channel cross-sections of 400×120, 600×180, 800×240, and 1200×360µm were fabricated to assess reliable printing and clearing of enclosed channels; the smallest consistently printable geometry was then selected for downstream numerical screening and experiments (Results). For separation studies, the turn number was varied between N=4 and N=8, and particle diameters were selected as $d_{S}=6\;µm$ (small/background, fixed) and $d_{L}=12$, 18, and 24µm (large/target).

The present device design was derived from a previously PDMS-optimized spiral inertial microfluidic platform, but its translation into an enclosed monolithic DLP-printed format required constrained re-optimization rather than direct replication. The final geometry was selected by considering both fabrication feasibility and the hydrodynamic conditions required for inertial focusing. In particular, the design process accounted for the coupled roles of channel aspect ratio, Reynolds number, Dean number, pressure drop, and wall shear stress. During optimization, the operating conditions were gradually increased such that the Reynolds number exceeded 100, while the corresponding Dean number remained within an approximate range of 20–80, which is widely regarded as favorable for inertial focusing in curved microchannels [27]. In this way, the final printed design was chosen not simply as the smallest printable geometry but as the smallest enclosed and printable geometry that could still operate within a hydrodynamically meaningful regime for stable inertial separation without excessive pressure drop or shear stress.

### 2.3. 3D Printing of Closed-Channel Devices

Closed-channel spiral devices were fabricated using a DLP UV projector-based microfluidic 3D printer (ProFluidics 285D; CADworks3D Microfluidics, Concord, ON, Canada; firmware v4.3.6 Rev0). Parts were prepared in ProFluidics Slicer (v6.4.4.t12; CADworks3D Microfluidics, Concord, ON, Canada) and printed using Clear Microfluidics Resin (V7.0a; CADworks3D Microfluidics, Concord, ON, Canada), providing a dynamic XY pixel resolution of 28.5 µm. Unless otherwise noted, printing was performed with a 50 µm layer thickness (Z resolution), 5 s exposure per layer, and no support structures. The device was oriented with the largest flat surface on the build plate, and printing was carried out under ambient conditions of 20–25 °C and 40–60% relative humidity.

After printing, the devices were post-processed in three steps: washing, UV curing, and inspection/assembly. First, printed parts were immersed in isopropyl alcohol (IPA) and gently agitated for 2–3 min. A fine syringe was then used to flush the enclosed channels and remove trapped resin. During manufacturability screening, additional clearing and flushing steps were performed as needed until a continuous flow path was obtained, defined by the absence of visible blockage and stable through-flow under syringe-pump actuation. The devices were then UV-cured for 20 min in a dedicated curing chamber (CureZone; CADworks3D Microfluidics, Concord, ON, Canada). The curing was performed from both sides to improve polymerization of the channel walls. Finally, the printed devices were examined under a microscope to confirm clear, undistorted channel walls and ports, and the port interfaces were lightly sanded before attaching Luer or tubing adapters.

**Figure 2:**
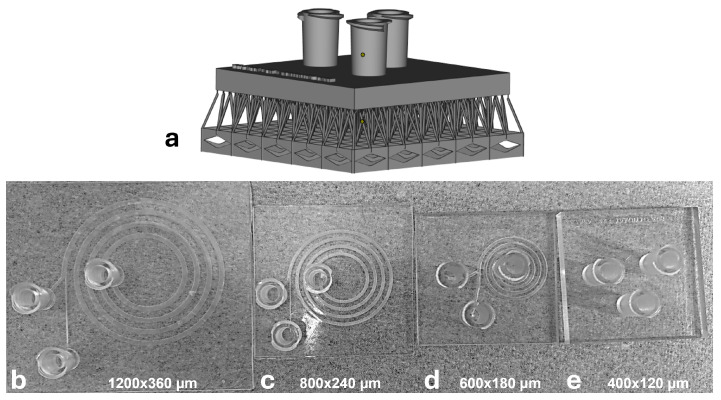
3D-printing trials and representative fabricated devices across channel cross-sections. (**a**) CAD rendering of the printed device with support structures. (**b**–**e**) Fabricated devices for channel cross-sections of 1200×360, 800×240, 600×180, and 400×120µm, respectively, illustrating the progressive decrease in printability and channel clearance quality with reducing cross-sectional dimensions.

Under this manufacturability screening (Figure 2), the 400×120µm and 600×180µm enclosed channels could not be cleared reliably, with blockage most frequently observed near the inner-outlet region. In contrast, the 800×240µm and 1200×360µm devices were printed and cleared reproducibly and were therefore used for downstream numerical screening and experiments.

### 2.4. Surface Roughness Characterization

**Figure 3:**
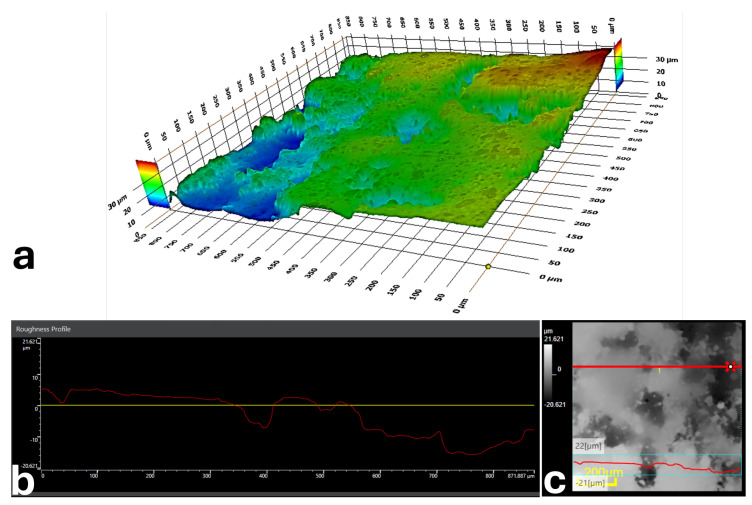
Representative surface roughness characterization of the printed device. (**a**) 3D Mapping of the surface; (**b**) roughness profile; (**c**) roughness profile location on the surface.

To document printing-induced surface texture, representative roughness measurements were acquired from printed channel surfaces. Because of the very small enclosed channel dimensions, direct sectioning of the printed channel introduced cutting-related defects on the exposed internal surface, preventing reliable quantitative roughness measurements from inside the channel. Therefore, the roughness information presented here was obtained from the outer printed surface of the structure to provide a representative indication of the printed bottom and sidewall characteristics of the channel. These observations are intended to offer qualitative insight into the printed surface morphology rather than an exact quantitative roughness analysis of the inner channel surface. The arithmetic mean roughness was $R_{a}\approx 2.4\;µm$ (Figure 3), reported here as a manufacturing descriptor for the additively manufactured devices used in this study.

### 2.5. Numerical Modeling

Three-dimensional numerical simulations were carried out in COMSOL Multiphysics (v5.4; COMSOL Inc., Stockholm, Sweden) to calculate the steady-state laminar flow field and predict particle trajectories within the spiral microchannel. The spiral geometry was first designed in SolidWorks and then imported into COMSOL for meshing and simulation.

The working fluid was modeled as an incompressible Newtonian fluid under laminar-flow conditions. No-slip boundary conditions were imposed on all channel walls. Particle trajectories were computed using a Lagrangian particle-tracing approach with one-way coupling, in which the flow field affects particle motion while particle feedback on the fluid is neglected. This assumption is appropriate for dilute suspensions. Polystyrene particles were assigned a density of $\rho_{p}=1050\;\mathrm{kg}/m^{3}$, with particle diameters of $d_{S}=6\;µm$ for the small/background particles and $d_{L}=12$, 18, and 24µm for the large particles.

At the channel inlet, the desired volumetric flow rate was prescribed, while both outlets were defined at zero gauge pressure. The steady-state flow field was obtained using the GMRES (Generalized Minimal Residual) iterative solver. Once a converged flow solution was obtained, particle trajectories were computed in a post-processing step using the same converged velocity field. Simulation post-processing included loop-by-loop visualization of particle focusing behavior along the spiral channel, as well as outlet-based particle counting to determine the distributions collected at the inner and outer outlets.

The computational domain was discretized using a physics-controlled mesh consisting of approximately 76,000 volume elements. The mesh included tetrahedral elements in the bulk flow region and prismatic boundary-layer elements near the channel walls to better resolve near-wall velocity gradients. Mesh quality was evaluated using the skewness metric, yielding an average element quality of 0.46. Additional simulations were performed with different mesh densities to confirm that the reported results were not sensitive to the spatial discretization level.

### 2.6. Particle Preparation

Before each experiment, the device was sequentially rinsed with 70% ethanol, ultrapure water, and PBS working buffer. An inverted fluorescence microscope equipped with a CCD camera (Axiocam 705 mono, ZEISS, Oberkochen, Germany) was used for imaging. The acquired images and experimental data were processed and analyzed using ImageJ^®^ Fiji v.1.54f (Media Cybernetics, Silver Spring, MD, USA).

Fluorescent polystyrene microspheres were used as size-defined surrogates. Experiments were performed using $d_{S}=6\;µm$ (small/background) and $d_{L}=18\;µm$ (large/target) particles, both with a density of $\rho_{p}\approx 1050\;\mathrm{kg}/m^{3}$. Stock suspensions were diluted in PBS containing 0.1% Polysorbate 20 to reduce aggregation, yielding working concentrations of approximately $2.18\times 10^{6}\;\mathrm{particles}/\mathrm{mL}$ for the 18µm population and $1.17\times 10^{7}\;\mathrm{particles}/\mathrm{mL}$ for the 6µm population. Suspensions were vortex-mixed immediately prior to each run to ensure homogeneous distribution.

**Figure 4:**
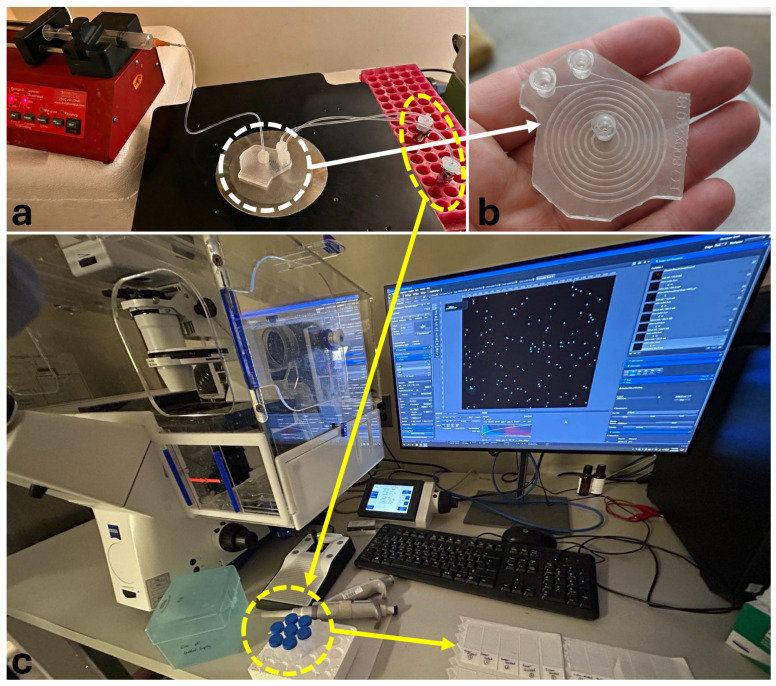
Experimental setup for particle-based validation. (**a**) Syringe pump connected to the spiral microfluidic device for controlled flow actuation; (**b**) close-up view of the 3D-printed closed-channel spiral microfluidic device; (**c**) full experimental setup including fluorescence microscope (ZEISS), imaging workstation, and inner/outer outlet collection reservoirs.

Flow was driven using a syringe pump (Braintree Scientific Inc., Braintree, MA, USA). Inner and outer outlet fractions were collected into separate reservoirs. Outlet samples were imaged using a ZEISS fluorescence microscope (ZEISS, Oberkochen, Germany) equipped with a monochrome camera (Axiocam 705 mono, ZEISS, Oberkochen, Germany) (Figure 4). Particles in the collected outlet samples were enumerated from fluorescence images using ImageJ/Fiji.

### 2.7. Experimental Setup

A syringe pump (Braintree Scientific Inc., Braintree, MA, USA) was used to deliver the sample at a flow rate of 6 mL/min. The syringe, with a diameter of 13.5 mm, was connected to the device inlet, while the two outlets were directed to separate collection reservoirs corresponding to the inner outlet and outer outlet.

For each experiment, the sample was introduced into the microfluidic device at the designated flow rate to maintain stable and continuous microflow. A 10 mL syringe was connected to the device using Tygon^®^ flexible tubing (Saint-Gobain, Northborough, MA, USA) with an internal diameter of 0.5 mm and a length of 30 cm.

### 2.8. Image-Based Counting

For particle quantification, fluorescence images collected from the inner and outer outlet reservoirs were analyzed using ImageJ/Fiji following a standardized counting procedure. The acquired images were first calibrated using the known image scale. Each image was then converted to 8-bit grayscale, and a consistent threshold was applied across all images to isolate fluorescent particle signals from the background. Particles were counted using the built-in Analyze Particlesfunction in ImageJ/Fiji, with size and circularity filters applied uniformly to all outlet images to exclude non-particle signals and aggregates. The resulting counts from three independent experimental runs were used for quantitative evaluation of separation performance.

Separation performance was quantified from outlet counts of large (L) and small (S) particles. Large-particle collection efficiency to the inner outlet and inner-outlet purity were defined as:

(6) $$\mathrm{Efficiency}=\frac{N_{L,\mathrm{inner}}}{N_{L,\mathrm{inner}}+N_{L,\mathrm{outer}}},$$

(7) $$\mathrm{Purity}=\frac{N_{L,\mathrm{inner}}}{N_{L,\mathrm{inner}}+N_{S,\mathrm{inner}}},$$

where $N_{L,\mathrm{inner}}$ and $N_{L,\mathrm{outer}}$ denote the counted numbers of large particles collected at the inner and outer outlets, respectively, and $N_{S,\mathrm{inner}}$ denotes the counted number of small particles collected at the inner outlet.

## 3. Results

### 3.1. Manufacturability-Driven Geometry Selection and Surface Quality

Direct translation of the previously demonstrated PDMS spiral geometry (400×120µm) into a monolithic, enclosed 3D-printed device was constrained by practical limitations of resin-based printing, particularly incomplete resin evacuation from long, fully encapsulated microchannels. During manufacturability screening across multiple cross-sections (Figure 2), the 400×120µm and 600×180µm enclosed channels could not be reliably cleared after printing, resulting in frequent blockage and unstable flow paths. Clogging was most often observed near the inner-outlet region, where local features are effectively narrow and the likelihood of trapped uncured resin is increased. In contrast, the 800×240µm and 1200×360µm designs were consistently printed and successfully cleared, enabling stable through-flow. Based on these results, the 800×240µm cross-section was selected as the smallest manufacturable geometry for subsequent numerical screening and experimental validation.

Because additively manufactured microchannels exhibit printing-induced surface texture, representative printed channel surfaces were characterized for roughness (Figure 3). The measured arithmetic average roughness was approximately $R_{a}\approx 2.4\;µm$, providing quantitative context for the surface condition of the printed devices used in this study.

### 3.2. Simulation-Guided Turn-Number and Particle-Size Screening

Numerical simulations were performed to assess how spiral development length (turn number) and target particle size influence outlet segregation in the selected 800×240µm geometry. To enable direct comparison across conditions, each simulated case used fixed particle populations: $N_{\mathrm{small}}=50$ small/background particles with $d_{S}=6\;µm$ and $N_{\mathrm{large}}=10$ large/target particles with $d_{L}=12$, 18, or 24µm. As part of the operating-condition screening, Reynolds and Dean numbers were also evaluated to confirm operation in the laminar regime and to guide selection of a suitable flow rate and turn number. This screening was particularly important because the larger 800×240µm cross-section reduces inertial lift compared with the PDMS-scale geometry, requiring re-optimization of residence length (turn number) and operating conditions to recover robust outlet segregation.

**Figure 5:**
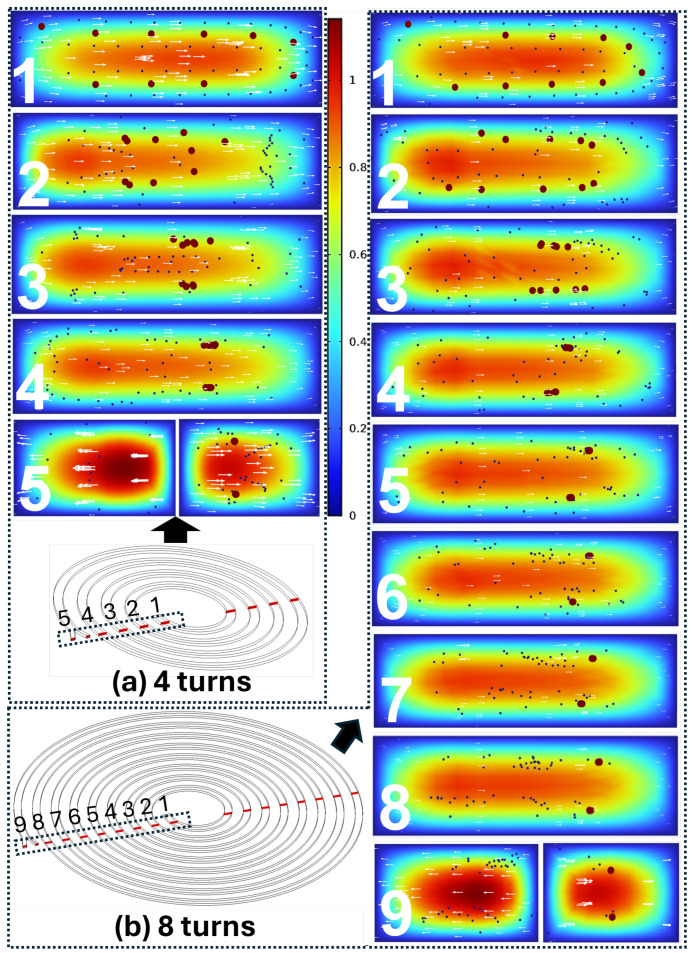
Loop-by-loop evolution of cross-sectional particle distributions and normalized velocity field for a representative condition ($d_{L}=18\;µm$, $d_{S}=6\;µm$): (**a**) 4-turn spiral and (**b**) 8-turn spiral.

A loop-by-loop visualization for a representative target size ($d_{L}=18\;µm$) compares 4-turn and 8-turn spirals in Figure 5 (left: 4 turns; right: 8 turns). In the 4-turn spiral, particles reach the outlets before clear segregation is established: although large particles begin to migrate toward the inner side, the small/background population remains broadly distributed and a substantial fraction still enters the inner outlet. Extending the spiral to 8 turns increases residence length and cumulative lateral migration, resulting in substantially improved outlet segregation.

**Figure 6:**
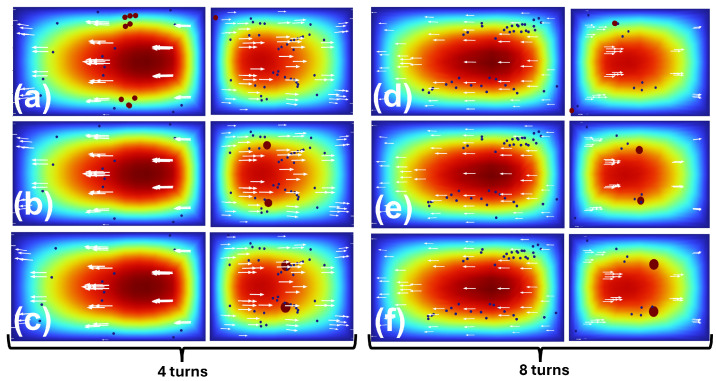
Outlet-level comparison of simulated particle distributions for spiral devices with different turn numbers and target particle diameters in the presence of a 6µm small/background population. The left column (**a**–**c**) shows the 4-turn spiral and the right column (**d**–**f**) shows the 8-turn spiral, each for $d_{L}=12$, 18, and 24µm from top to bottom.

Outlet-level comparisons for multiple target diameters are shown in Figure 6 (left: 4 turns; right: 8 turns). Across these conditions, the small/background population follows a similar overall trend, whereas large-particle displacement strengthens with increasing diameter. Quantitatively, the PDMS-scale reference case (400×120µm, 4 turns) yielded complete large-particle collection at the inner outlet (10/10; 100% efficiency). In contrast, for the 3D-printable 800×240µm geometry at 4 turns and $d_{L}=12\;µm$, large-particle collection dropped to 1/10 (10% efficiency). Intermediate turn numbers (5–7 turns) recovered large-particle collection to 5/10 (50% efficiency) for the screened $d_{L}=12\;µm$ condition. The 8-turn 800×240µm spiral achieved complete large-particle collection at the inner outlet (10/10; 100% efficiency) for $d_{L}=12$, 18, and 24µm, together with reduced small-particle leakage to the inner outlet compared to shorter spirals (Table 2).

In the present device, the operating condition was selected such that the Dean number was maintained around 20, which in turn kept the Reynolds number below 200. At higher flow conditions, the flow became less stable and particle trajectories were less well defined, consistent with prior studies showing that inertial focusing behavior in curved channels is strongly Reynolds-number-dependent and can be perturbed as Dean-coupled secondary flow becomes increasingly dominant.

### 3.3. Experimental Separation Performance of the 3D-Printed 8-Turn Device

**Figure 7:**
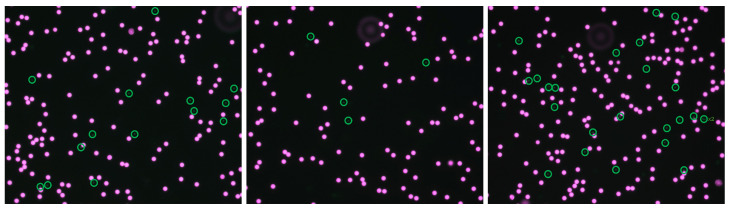
Representative fluorescence images used for experimental particle counting at the outlets for the 8-turn 800×240µm device ($Q=6\;mL\;\min^{-1}$, $d_{L}=18\;µm$, $d_{S}=6\;µm$). The inner outlet corresponds to the large-particle collection outlet.

Based on the simulation screening, the 8-turn 800×240µm device was selected for experimental validation. Experiments were conducted using fluorescent polystyrene particles with $d_{L}\approx 18\;µm$ (large/target) and $d_{S}\approx 6\;µm$ (small/background) at a flow rate of $Q=6\;mL\;\min^{-1}$. Representative fluorescence images used for outlet counting are shown in Figure 7. Particle counts collected from the inner and outer outlets were used to calculate large-particle collection efficiency and inner-outlet purity according to the definitions given above. The quantitative results from three independent runs are summarized in Table 3.

Overall, the monolithic 3D-printed closed-channel spiral device achieved an average large-particle collection efficiency of 84.1% and an average inner-outlet purity of 91.9% (Table 3). The nominal particle diameter used in the experiments was ≈18 µm, with an actual size distribution in the range of approximately 15–19 µm. Since inertial microfluidic separation is strongly dependent on particle size, this size variation may contribute to differences in efficiency between Comsol analysis and printed methods. The efficiency of 84.1% and purity of 91.9% demonstrate robust size-based segregation in a fully enclosed printed platform, despite manufacturability-driven scaling of channel cross-section and the presence of printing-induced surface texture.

## 4. Discussion

This work addresses the challenge of transferring an optimized spiral inertial microfluidic separator from a PDMS-based fabrication platform to a monolithic, fully 3D-printed, closed-channel format. In our earlier PDMS implementation, a smaller cross-section (400×120µm) enabled strong size-based separation after systematic optimization [18]. Here, the limiting factor was not the spiral separation principle itself, but the practical manufacturability of long, fully enclosed microchannels in resin printing, where reliable post-print clearing ultimately determines whether a design is functional. This distinction is well recognized in the 3D-printed microfluidics literature: nominal printer resolution does not directly translate to minimum functional enclosed channel dimensions because resin overcuring, trapped resin evacuation, and post-processing access dominate in sealed networks [28,29,30].

The significance of the present work lies not only in the reduced channel height, but also in the fact that successful fabrication and operation were achieved in a long, enclosed 8-turn spiral geometry rather than in a short or straight channel. Post-processing is inherently more difficult in such extended curved channels, where trapped resin is harder to evacuate and blockage becomes more likely, particularly near confined outlet regions, a limitation widely recognized in stereolithographic microfluidics [25]. This challenge is also consistent with prior reports on enclosed 3D-printed microchannels. For example, Castiaux et al. not only demonstrated PolyJet-printed enclosed microfluidic channels down to 125 × 54 µm, but also emphasized that support-material removal becomes difficult for channels with characteristic dimensions below approximately 200 µm and nearly impossible for designs containing turns or serpentines [31]. This further highlights why successful clearing of the present long enclosed spiral geometry is nontrivial. Thus, the novelty here is not simply the realization of a 240 µm enclosed channel, but the successful fabrication and clearing of such a channel in a functionally relevant long spiral geometry. This distinction becomes clearer when compared with prior high-resolution demonstrations. Gong et al. achieved long enclosed serpentine channels and extremely narrow features, but with a custom-built DLP-SLA platform and custom resin [23]. Kuo et al. demonstrated assembly-free enclosed 3D biomicrofluidic architectures with complex channel routing, but with characteristic channel heights generally larger than those used here [24]. In this context, the main contribution of the present study is to show that a meaningful inertial spiral separation geometry can be realized using a low-cost and accessible DLP platform, despite the combined constraints of small enclosed dimensions, long channel length, and difficult post-print clearing.

The fabrication trials conducted in this study directly confirmed this constraint. Although the PDMS-scale geometry (400×120µm) was tested in a closed-channel printed format, it could not be cleared reproducibly, and the 600×180µm design exhibited similar failure modes. Blockage occurred most frequently near the inner-outlet region, consistent with this zone being particularly sensitive to trapped resin and local geometric restrictions in enclosed layouts. In contrast, 800×240µm (and larger) channels were repeatedly printable and flushable, leading to the selection of 800×240µm as the smallest reliably functional cross-section in our workflow. This outcome aligns with comparative studies and reviews emphasizing that the practical lower limit for enclosed channels is set by the coupled effects of print physics and cleaning feasibility rather than by XY pixel size alone [28,29,30]. It is also consistent with the broader observation that channel surface roughness and limited print resolution can disturb laminar flow profiles and degrade focusing efficiency in inertial microfluidic devices [32]. In the context of inertial microfluidics, such manufacturing constraints are particularly important because spiral devices rely on long, continuous flow paths; even small local constrictions or partial occlusions can disrupt outlet partitioning.

From a transport-physics standpoint, scaling the channel cross-section modifies the relative balance between inertial lift and Dean-flow-driven transverse drag over a given residence length. Inertial microfluidics operates in laminar yet inertia-relevant regimes where lift forces drive size-dependent migration to equilibrium positions [6,7,10]. In curved channels, Dean vortices introduce secondary flows that interact with inertial lift and can accelerate or reshape lateral migration, enabling compact separation in spirals [12,13]. In our manufacturable 800×240µm geometry, Reynolds and Dean number calculations were used as a sanity check to verify that operation remained laminar while still lying within the inertial focusing regime. In the present device, the operating condition was selected to maintain the Dean number at around 20, thereby keeping the Reynolds number below 200. Under these constraints, the design effort focused on recovering robust separation in a larger cross-section, where lift-driven migration can weaken for a given particle size and development length. Rather than treating this as a drawback, we used it as an explicit design driver: manufacturability sets the minimum cross-section, and hydrodynamic performance is then recovered by re-optimizing the development length and operating conditions. This approach is conceptually consistent with recent work demonstrating that large-dimension spiral channels can still achieve effective inertial focusing when secondary flow patterns are carefully controlled through geometry optimization [33].

The simulation screening supports this mechanism clearly. In the 800×240µm channel, the 4-turn spiral was insufficient for the smallest tested large particles (D12), yielding low collection at the inner outlet. While D18 and D24 showed complete large-particle collection even at 4 turns, a substantial number of small/background particles still entered the inner outlet, reducing inner-outlet purity. This highlights a practical point that is sometimes underappreciated in spiral separators: high collection efficiency for the target population does not guarantee clean fractionation if the background population has not narrowed to a well-separated streamline at the outlet split, and outlet performance can be sensitive to small shifts in equilibrium position and flow partitioning [6,12,13]. Increasing the turn number (5–7 turns) improved performance for the challenging D12 case, indicating a transition toward stable partitioning as residence length increases. The 8-turn design provided the most robust simulated outcome across diameters (D12, D18, D24), simultaneously preserving large-particle collection at the inner outlet and reducing small-particle crossover. The loop-by-loop cross-sectional visualization (4 turns vs. 8 turns) reinforces the same interpretation: in shorter spirals, particles reach the bifurcation before a stable lateral separation fully develops, whereas longer spirals provide sufficient development length for focused distributions to mature prior to splitting.

Experimental validation was performed under the operating point most relevant to our printed platform: fluorescent polystyrene particles with $d_{L}\approx 18\;µm$ and $d_{S}\approx 6\;µm$ at $Q=6\;mL\;\min^{-1}$ in the 8-turn 800×240µm printed device, where the inner outlet is designated as the large-particle collection outlet. The measured average collection efficiency (84%) and inner-outlet purity (92%) confirm that manufacturable, fully enclosed printed spirals can retain strong size-based separation performance when geometry is re-optimized around the constraints of printing and clearing. The remaining gap relative to idealized simulation outcomes is expected for monolithic printed channels and can plausibly be attributed to non-idealities that are minimal in PDMS casting, including printing-induced surface roughness and local dimensional deviations that broaden focused streams, slight flow-split sensitivity at the outlet junction, and occasional partial occlusions or microbubble trapping. Such effects are repeatedly emphasized in studies comparing printed microfluidics to conventional fabrication, where functional performance is governed by surface condition, dimensional fidelity, and post-processing quality beyond nominal resolution [28,29,30]. The numerical analysis assumed monodisperse 18 µm particles, whereas the experimental particles exhibited a broader size range of approximately 15–19 µm, although the sample was denoted as ∼18 µm because its dominant fraction was centered near this value. This particle-size variation, together with fabrication-related non-idealities such as wall roughness, dimensional deviations, and stair-stepping, likely contributed to the discrepancy between the idealized simulation and the experimental results. Nevertheless, considering the manufacturability constraints and the intended cost–performance balance of the platform, the experimentally obtained separation efficiency remained satisfactory for the scope of this study.

When contextualized with prior 3D-printed spiral and inertial microfluidics studies, our findings fall within a realistic and consistent range. Enders et al. demonstrated a 3D-printed spiral separation device and highlighted that practical channel dimensions (including approximately ∼200 µm-scale heights) are selected to match what can be produced and cleared reliably, reinforcing that manufacturability thresholds directly shape spiral design choices in practice [34]. Similarly, Sierra Agudelo et al. emphasized a low-cost 3D-printed inertial microfluidic workflow and reported that larger channel dimensions are often selected in part due to printer and process capabilities, consistent with a manufacturing-first design rationale [22]. More broadly, 3D printing studies focused on inertial microfluidic devices show that performance is achievable but depends strongly on the chosen printing platform, resin, cleaning strategy, and the extent to which geometry is co-optimized for both hydrodynamics and fabrication [28,35]. Internal surface roughness and printing-related geometric deviations may affect the effective channel dimensions and local flow behavior. As noted in recent fabrication reviews, high surface roughness in additively manufactured microchannels can disturb laminar velocity profiles and degrade inertial focusing performance, further motivating the use of the smallest reliably clearable geometry rather than the smallest nominally printable one [32]. However, due to the very small enclosed channel size, reliable quantitative characterization of the inner channel roughness could not be performed without introducing cutting-related damage. This remains a limitation of the current study and may partially contribute to deviations in experimental performance. Together, these works support the broader implication that effective printed spiral separators are typically obtained by co-optimizing cross-section, development length, and operating conditions around what can be fabricated and cleaned reproducibly, rather than attempting a direct replication of PDMS-optimized minimum cross-sections.

Overall, the results support a manufacturing-first optimization pathway for translating spiral inertial microfluidics to additive manufacturing. In our case, insisting on the PDMS-scale 400×120µm enclosed geometry was not viable due to clearing failures, whereas the 800×240µm cross-section enabled reproducible fabrication and systematic turn-number optimization. The practical design lesson is therefore twofold: it first identifies the smallest enclosed channel that is consistently printable and cleanable in the chosen workflow, and then recovers separation robustness by tuning development length (turn number) and operating conditions within laminar, inertia-relevant regimes [6,7,10]. Future improvements can focus on reducing residual crossover and run-to-run variability by refining outlet hydraulic resistance ratios, improving clearing protocols and print orientation to reduce resin entrapment probability near the inner outlet, and exploring process/resin options that improve dimensional fidelity and surface quality while maintaining optical transparency [28,29,30].

## 5. Conclusions

This study demonstrates a manufacturing-informed translation of spiral inertial microfluidic separation from a PDMS-optimized baseline to a monolithic, fully 3D-printed, closed-channel device. Printing trials showed that enclosed 400×120µm and 600×180µm channels could not be cleared reproducibly due to trapped resin and frequent blockage, particularly near the inner-outlet region. In contrast, 800×240µm and 1200×360µm channels were consistently printable and flushable, and 800×240µm was selected as the smallest reliably functional cross-section in our workflow.

To recover robust size-based segregation in the scaled geometry, the spiral development length and operating regime were re-optimized using numerical screening and Reynolds/Dean number considerations to remain within a laminar yet inertia-relevant flow range. Turn-number studies showed that short spirals provide insufficient migration length in the larger cross-section, whereas increasing the spiral to 8 turns yields the most consistent outlet partitioning across tested target sizes. Experimental validation of the 8-turn 800×240µm device at $Q=6\;mL\;\min^{-1}$ using fluorescent polystyrene particles ($d_{L}=18\;µm$, $d_{S}=6\;µm$) confirmed robust separation, achieving an average large-particle collection efficiency of ∼84% and inner-outlet purity of ∼92%.

Although the measured performance is modestly reduced compared with the previously reported PDMS baseline [18], the present results establish a practical and scalable route for manufacturing closed-channel spiral separators as monolithic printed parts. Overall, the work provides an engineering framework in which printability and post-print clearing are treated as primary constraints, and separation performance is recovered through geometry and residence-length re-optimization. Future improvements can focus on outlet resistance tuning, enhanced clearing protocols, and process/resin choices that improve dimensional fidelity and surface quality to further reduce small-particle crossover and improve run-to-run repeatability.

## Figures and Tables

**Table 1:** Design variables used for manufacturability screening, numerical modeling, and experiments.

Parameter	Values Used in This Study
Channel cross-section, W×H (µm)	400×120, 600×180, 800×240, 1200×360
Spiral turn number, *N* (turns)	4–8
Small particle diameter, $d_{S}$ (µm)	6 (fixed)
Large particle diameter, $d_{L}$ (µm)	12, 18, 24

**Table 2:** Simulation-derived outlet particle counts for turn-number and particle-size in the 800×240µm channel.

Turns	*W* (μm)	*H* (μm)	*Q* (mL min^−1^)	$d_{L}$(μm)	Large_inner_	Large_inner_	Small_inner_	Small_outer_
4	400	120	3	12	10	0	0	50
4	800	240	6	12	1	9	36	14
4	800	240	6	18	10	0	36	14
4	800	240	6	24	10	0	37	13
5	800	240	6	12	5	5	33	17
6	800	240	6	12	5	5	19	31
7	800	240	6	12	5	5	14	36
8	800	240	6	12	10	0	9	41
8	800	240	6	18	10	0	8	42
8	800	240	6	24	10	0	9	41

**Table 3:** Outlet particle counts and calculated performance metrics for the 8-turn 800×240µm 3D-printed device at $Q=6\;mL\;\min^{-1}$ using fluorescent polystyrene particles ($d_{L}=18\;µm$, $d_{S}=6\;µm$).

$N_{L,\mathrm{inner}}$	$N_{S,\mathrm{inner}}$	$N_{L,\mathrm{outer}}$	$N_{S,\mathrm{outer}}$	Efficiency	Purity
149	14	27	1375	0.847	0.914
107	4	20	894	0.843	0.964
177	24	35	1620	0.835	0.881
Average	–	–	–	0.841	0.919

## Data Availability

Data are available from the authors on request.
